# *UGT1A1* Genetic Variations and a Haplotype Associated with Neonatal Hyperbilirubinemia in Indonesian Population

**DOI:** 10.1155/2018/9425843

**Published:** 2018-01-23

**Authors:** Dewi A. Wisnumurti, Yunia Sribudiani, Robert M. Porsch, Ani M. Maskoen, Lola I. Abdulhamied, Sri E. Rahayuningsih, Eni K. Asni, Frank Sleutels, Christel E. M. Kockx, Wilfred F. J. van Ijcken, Abdurachman Sukadi, Tri H. Achmad

**Affiliations:** ^1^Neonatology Subdivision, Pediatric Department, Arifin Achmad General Hospital, Pekanbaru, Indonesia; ^2^Department of Biochemistry and Molecular Biology, Faculty of Medicine, Universitas Padjadjaran, Bandung, Indonesia; ^3^Department of Psychiatry, Li Ka Shing Faculty of Medicine, The University of Hong Kong, Pokfulam, Hong Kong; ^4^Department of Epidemiology and Biostatistics, Faculty of Medicine, Universitas Padjadjaran, Bandung, Indonesia; ^5^Cardiology Subdivision, Pediatric Department, Dr. Hasan Sadikin Hospital, Bandung, Indonesia; ^6^Department of Biochemistry, Faculty of Medicine, Universitas Riau, Riau, Indonesia; ^7^Erasmus Center for Biomics, Erasmus MC, Rotterdam, Netherlands; ^8^Neonatology Subdivision, Pediatric Department, Dr. Hasan Sadikin Hospital, Bandung, Indonesia

## Abstract

Neonatal hyperbilirubinemia (NH) is a common finding in newborn babies in Indonesia. Common and rare variants of* UGT1A1* have been known to contribute to NH etiology. This study aims to identify* UGT1A1* genetic variation and haplotype associated with NH in Indonesian population. DNA was isolated from 116 cases and 115 controls and a targeted-deep sequencing approach was performed on the promoter, UTRs, and exonic regions of* UGT1A1*. Determining association of common variants and haplotype analysis were performed using PLINK and Haploview. Ten and 4 rare variants were identified in cases and controls, respectively. The* UGT1A1* rare variants frequency in cases (5.17%) was higher than that in controls (1.7%). Four of those rare variants in cases (p.Ala61Thr, p.His300Arg, p.Lys407Asn, and p.Tyr514Asn) and three in controls (p.Tyr79X, p.Ala346Val, and p.Thr412Ser) are novel variants. The frequencies of p.Gly71Arg, p.Pro229Gln, and TA_7_ common variants were not significantly different between cases and controls. A haplotype, consisting of 3 major alleles of 3′ UTRs common variants (rs8330C>G, rs10929303C>T, and rs1042640C>G), was associated with NH incidence (*p* = 0.025) in this population. Using targeted-deep sequencing and haplotype analysis, we identified novel* UGT1A1* rare variants and disease-associated haplotype in NH in Indonesian population.

## 1. Introduction

Jaundice is a common physiological phenomenon in neonates as it occurs almost in 60% of healthy term newborns [1, 2]. Neonatal hyperbilirubinemia (NH) is a condition when jaundice with the serum total bilirubin (STB) levels is above the 95th percentile for age in hours [3]. In the most severe cases, NH leads to chronic bilirubin encephalopathy (kernicterus) which is characterized by severe athetoid cerebral palsy, sensory hearing loss, dental-enamel dysplasia, paralysis upward gaze, and mortality. Neonatal hyperbilirubinemia occurs due to the imbalance between production and elimination of bilirubin [2, 4]. One of the important processes in bilirubin elimination is glucuronic acid conjugation to bilirubin. Conjugated bilirubin is more polar and easier to eliminate compared to unconjugated bilirubin. The conjugation process occurs in the hepatocyte and is catalyzed by UDP-glucuronosyltransferase enzyme which is encoded by* UGT1A1 *gene [2, 4]. The prevalence of NH varies in different populations with the highest being in the Navajo Indian, Greek, and East Asian such as Japanese and Korean [4]. The previous study showed that the risk of experience hyperbilirubinemia is 12.5-fold higher than that in controls population if a previous sib had STB levels higher than 15 mg/dL [4, 5]. Furthermore, it has been reported that there was a high concordance of STB levels among identical twins in the European and Chinese. These findings suggest that a genetic component might contribute to the development of NH [4].

It has been reported that* UGT1A1 *rare and common variants were associated with NH in different populations [6–11]. It has been known also that* UGT1A1 *variants were underlying cause of prolonged unconjugated hyperbilirubinemia associated with breast milk in Japanese population [12]. These show that combination of genetic and nongenetic factors contributes to the development of the disease. More than 130* UGT1A1* variants have been reported causing Gilbert's syndrome and Crigler-Najjar syndrome type 1 (CN1) and type 2 (CN2), which are characterized by inherited nonhemolytic unconjugated hyperbilirubinemia. The insertion of TA sequence in the TATAA box of the promoter region (TA_7_) had been reported to be associated with NH in North Indian population [13]. The meta-analysis performed by Long et al., 2011, showed that p.Gly71Arg common variant was reported to be associated with NH in Asian but not in Caucasian population [14]. However, the recent meta-analysis study performed by Yu et al., 2015, from 32 studies with total 6520 participants, showed that the TA_7_ and p.Gly71Arg variants also significantly increased the risk of NH in both Caucasian and Asian population [15]. In this study we aim to clarify* UGT1A1* genetic variation and haplotypes associated with NH in Deutromalay, one of the major ethnic groups in Indonesia.

## 2. Materials and Methods

### 2.1. Patients, Parents, and Controls

One hundred sixteen healthy term neonates with hyperbilirubinemia and 115 without hyperbilirubinemia from Deutromalay population in Indonesia were enrolled in this study. Diagnosis of hyperbilirubinemia was based on the American Academic of Pediatrics (AAP) criteria where cases are defined as neonates whose serum total bilirubin (STB) levels were above the 95th percentile of the corresponding age group. All cases and controls neonates were single birth. We excluded patients with hemolytic anemia, maternal diabetes, cephalohematoma, neonatal sepsis, incompatibility of ABO, and Rhesus blood grouping between mother and the babies and other congenital diseases which would affect the level of bilirubin in the serum. Written informed consent was obtained from parents and the study was approved by the Ethic Committee of the Faculty of Medicine, Universitas Padjadjaran, Bandung, Indonesia.

### 2.2. DNA Isolation

Genomic DNA was extracted from one-milliliter peripheral blood leukocytes using Genomic DNA isolation kit (Roche Life Sciences) in MagNA Pure LightCycler 32 instrument (Roche Life Sciences, Pleasanton, USA) according to the manufacturer's protocol.

### 2.3. Deep-Targeted Next-Generation Sequencing (NGS) and Data Analysis

The* UGT1A1* gene was enriched with the* TruSeq Custom Amplicon *assay (Illumina, San Diego, USA). The oligos used in this assay were designed using* Design Studio *(Illumina, San Diego, USA). Amplicons cover all exons, padded with 10 base pairs (bp) on each end and 250 bp of the promoter region. The amplicons were sequenced using paired-end sequencing of 2 × 250 bps on a MiSeq (Illumina, San Diego, CA, USA). Data processing was performed with the MiSeq Reporter software (Illumina, San Diego, CA, USA) that performs the alignment against the human reference genome build 19 (hg19) with the Burrows-Wheeler Aligner (BWA) [16]. Subsequently, genetic variants were called with the genome analysis toolkit (GATK) [17]. Identified variants were filtered for known variants in dbSNP138 database using SeattleSeq software (http://snp.gs.washington.edu/SeattleSeqAnnotation138/). Variants not known in dbSNP138 or minor allele frequency (MAF) < 0.01 were categorized as rare variants and variants with MAF ≥ 0.01 were categorized as common variants.

### 2.4. Association Analysis of Identified Common Variants

The association analysis of common variants was performed using PLINK [18]. We excluded variants from the association test that were either monomorphic, had a minor allele frequency (MAF) < 0.05, or were missing in 95% of all subjects. We used the False Discovery Rate (FDR) to correct *p* values for multiple testing [19].

### 2.5. Haplotype Analysis

Analysis of linkage disequilibrium (LD) and haplotype structure mapping were calculated and graphically displayed using Haploview software developed by Broad Institute in Cambridge, USA (https://www.broadinstitute.org).

### 2.6. Validation of Rare Variants

**Figure 1:**
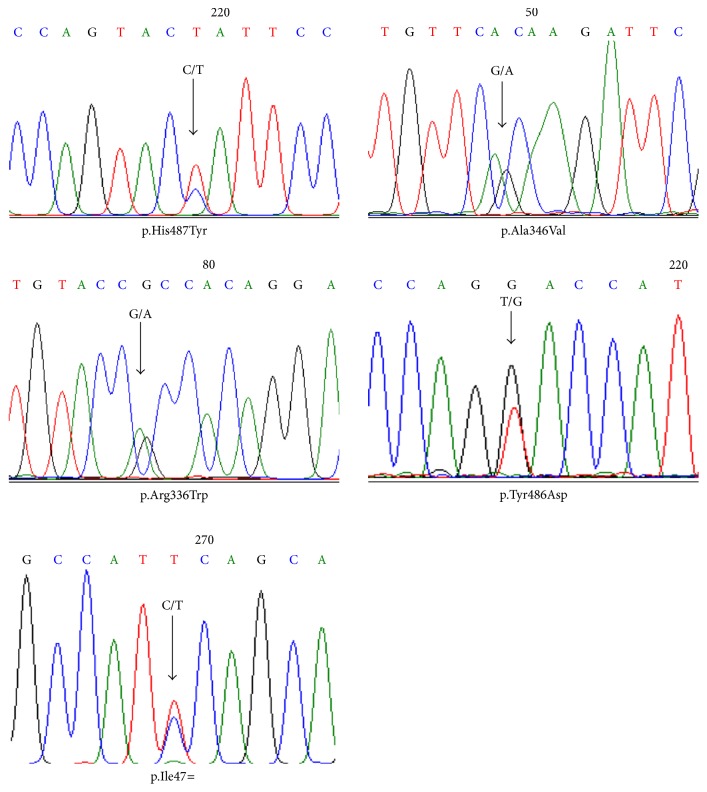
Validation of* UGT1A1* rare variants using Sanger Sequencing.

Polymerase Chain Reaction (PCR) and Sanger Sequencing were performed to validate the rare variants identified by deep-targeted next-generation sequencing method. The validation step was performed only for few variants in which DNA samples were still available (Figure 1). Primers used for PCR are presented in Table S1. A mixture of 50–100 ng of DNA, 25 *μ*l of 2x KAPA2G Fast PCR Kits (KAPA Biosystems, Wilmington, Massachusetts, USA), and 10 *μ*M of each forward and reverse primer were prepared in a total volume of 50 *μ*l. All exons containing identified* UGT1A1* rare variants were amplified by touch-down PCR with the annealing temperature ranging from 68°C to 58°C in the first 10 cycles and 58°C for the last 25 cycles. PCR products were gel purified and sequencing was performed using Big Dye Terminator v3.1 Kit (Applied Biosystems, Foster City, USA) on an ABI 3130XL automated sequencer.

### 2.7. In Silico Analysis

The pathogenicity of identified rare variants was predicted with three online software programs: PolyPhen-2 (http://genetics.bwh.harvard.edu/pph2/), SIFT (http://sift.jcvi.org/), and Mutation Taster® (http://www.mutationtaster.org/). Those three software packages use different algorithmic models as described by Dong et al., 2014 [20].

### 2.8. Statistical Analysis

Statistical analysis was performed using R version 3.3.1 and GraphPad. Differences in proportion of rare and common variants frequencies between cases and controls were analyzed using Fisher's exact test. Differences of serum total bilirubin (STB) levels according to genotypes were analyzed using one-way ANOVA. In all analyses, *p* value < 0.05 was considered statistically significant.

## 3. Results

### 3.1. Patients Characteristics

Characteristics of neonates and their mothers enrolled in this study are presented in Tables S2 and S3, respectively. The mean of birth weight was 3,125 ± 345 g in cases and 3,138 ± 371 g in the controls. The results of ABO and Rhesus blood grouping incompatibility between the mothers and the neonates were negatives in all samples. The results of blood analyses are presented in Table S4. There was no statistical difference in the type of feeding methods. The age of the mother and the number of parities between the mothers of neonates in cases and control groups were also not significantly different. The majority of the mothers from both groups had Cesarean delivery. Taken together, we have collected an appropriated cohort for studying the involvement of common and rare variants in the development of NH in Deutromalay.

### 3.2. Identified Rare and Common Variants

**Figure 2:**
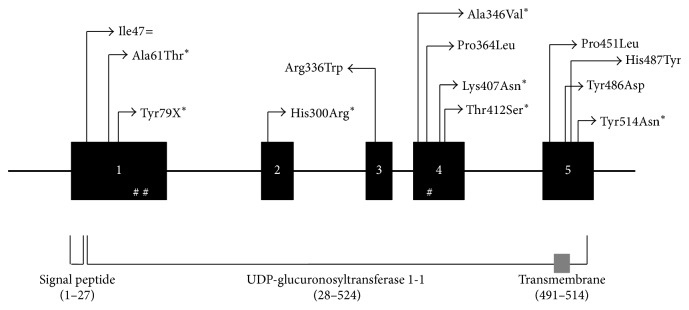
Schematic of* UGT1A1* (NM_000463) common and rare variants identified in infants with and without hyperbilirubinemia in Deutromalay ethnic group. Exons are presented in numbered black boxes. Intron, 5 UTR, and 3 UTR are presented in solid bar, the glycosylation sites (amino acids 102, 295, and 347) are presented in #, and novel mutations are marked with the *∗* sign. UGT1A1 enzyme has 3 domains: signal peptide domain (amino acid 1–27), UDP-glucuronosyltransferase 1-1 domain (amino acids 28–524), and transmembrane domain (amino acids 491–514) which is overlapping with second domain (grey box).

The statistics of the next-generation sequencing result is presented in Table S5. The majority of the identified variants (88 and 89%) were Single Nucleotide Polymorphism (SNP) in both cases and controls, respectively. The rest of identified variants were insertions and deletions (indels) which account for 11 and 12% in controls and cases, respectively. Up to 92% of the SNPs were located in the 3′ UTRs region and the remaining SNPs were located in the coding region. In total we identified 10 and 4 different heterozygous rare variants in 12 cases (5.17%) and 4 controls (1.7%), respectively. Four of those rare variants in cases (p.Ala61Thr, p.His300Arg, p.Lys407Asn, and p.Tyr514Asn) and three in controls (p.Tyr79X, p.Ala346Val, and p.Thr412Ser) were never reported before (novel variants) (Table 1). Those rare variants identified in cases and controls were scattered through all exons (Figure 2). Most of the identified rare variants were missense variants, whereas one of them was a stop-gain variant (p.Tyr79X) and one silent variant (p.Ile47=). Two common variants located in the coding region (p.Gly71Arg and p.Pro229Gln) were identified. The frequency of p.Gly71Arg was 6.89% and 4.78% in cases and controls, respectively. The frequency of p.Gly71Arg in this population was higher than that in the Javanese population from Central Java, Indonesia (1.5%), and in well-term infants from Malays in Singapore (4%) [8, 21]. The frequency of p.Pro229Gln was 4.74% and 2.60% in cases and controls, respectively. The frequency of p.Pro229Gln in controls in this population was lower than that in well-term infants from Malays (6%) but higher than that in Chinese population (2%) in Singapore [8]. p.Gly71Arg variant was reported to be associated with NH in Taiwanese, Japanese, and Malaysian Chinese population [22–25], while the p.Pro229Gln was reported to be identified in infants and adult with hyperbilirubinemia in Japanese and Chinese population, respectively [26]. In this study, the Odds Ratio (OR) of p.Gly71Arg and p.Pro229Gln was 1.47 and 1.59, respectively; however none of the *p* values was significant, showing that none of those common variants was associated with NH (Table 1). A heterozygous rare variant p.Tyr486Asp was identified in an infant with hyperbilirubinemia in this study. Eleven and 12% of the identified variants in cases and controls, respectively, were indels; however those indels consist of only insertion and deletion of TA sequences in the TATAA box region of the* UGT1A1* promoter (rs8175347). The insertion and deletion of TA were common variants as their MAF in the population is higher than 0.01. Three different genotypes of TA repeats in this region were identified: TA_6_/TA_6_ (wild type), TA_6_/TA_5_, and TA_6_/TA_7_ (Table S6). It has been known that the insertion of TA sequence in this region (TA_7_ allele) could reduce the expression of* UGT1A1* gene leading to insufficiency of UGT1A1 enzyme, while the TA_5_ allele could increase the expression of* UGT1A1* [27]. Insertion of TA sequence, either TA_7_ or TA_8_, has been reported to be associated with NH in different population [8, 9, 28]. The frequency of the TA_7_ variant observed in this study was 10.8% and 9.5% in cases and controls, respectively. These numbers are quite similar to those reported in infants with NH in the Chinese (11.5%) and Malay (10.7%) population but lower than that in Indian population (37.1%) in Singapore [8]. The OR of TA_7_ alleles in this study is 1.20 with 95% CIs: 0.65–2.22 and *p* value: 0.57 (Table S6). Validation of rare variants by Sanger Sequencing is performed in this study only for those whose DNA of the subjects were still available; those were p.His487Tyr, p.Ala346Val, p.Arg336Trp, p.Tyr486Asp, and p.Ile47= (Figure 1).

### 3.3. In Silico Analysis

In silico analysis using PolyPhen-2, SIFT, and Mutation Taster predicted that most of the identified rare variants in the coding regions in both cases and controls are damaging variants based on at least two different software packages (Table 2).

### 3.4. Association Analysis of Common Variants

In total 51 different variants located in coding and noncoding regions were identified in both cases and controls. Out of those 51 variants there were only 5 Single Nucleotides Polymorphisms (SNPs) which were included in the association test using PLINK, since the remaining variants were either monomorphic, had a MAF < 0.05 (rare variants), or were missing in 95% of all subjects. After adjusting for multiple testing none of the 5 tested SNPs remains significant at a threshold of *p* value = 0.05 (Table 3).

### 3.5. Haplotype Analysis

**Figure 3:**
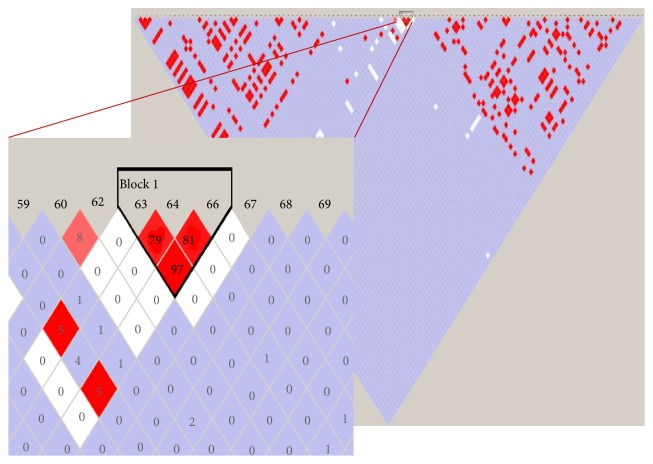
Linkage disequilibrium (LD) structure across* UGT1A1 *in Deutromalay population in Indonesia. SNPs rs8330, rs10929303, and rs1042640 are in the linkage disequilibrium with *r*^2^ = 0.97 (block 1). The CCC haplotype is associated with neonatal hyperbilirubinemia with *p* value of 0.0025 (Table 4).

To further analyze the effect of the combination of SNPs in* UGT1A1* on the development of NH in this population, we performed haplotype analysis using Haploview. Three linkage disequilibrium blocks with *r*^2^ between 0.8 and 0.97 were identified (Figure 3). The block with the strongest linkage disequilibrium (*r*^2^ = 0.97) consists of three SNPs located in the 3′ UTRs region; those were rs8330 (C>G), rs10929303 (C>T), and rs1042640 (C>G). The same set of SNPs appeared in the results of association analysis of common variants using PLINK (Table 3). CCC haplotype (major alleles/wild type) was significantly associated with NH (*p* value = 0.025). The other two haplotypes (minor alleles: GTG and CTC) were more present in the controls than in the cases and only the frequency of CTC haplotype was significantly different between cases and controls (*p* = 0.0083) (Table 4). This suggests that the CTC haplotype might have a protective effect on the development of NH. However, these results should be viewed with caution given the low sample size.

### 3.6. Comparison of STB Levels according to Genotypes

**Figure 4:**
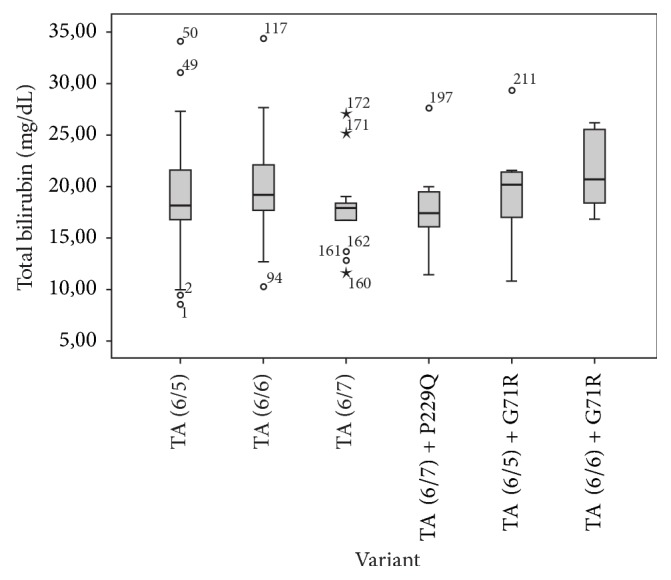
Total bilirubin levels of infants in cases group carrying different* UGT1A1 *common variant or combination of several common variants (TA_6_TA_5_, TA_6_TA_6_, TA_6_TA_7_, TA_6_TA_7_ + p.Pro229Gln, TA_6_TA_5_ + p.Gly71Arg, and TA_6_TA_7_ + p.Gly71Arg). There was no significant difference of serum total bilirubin expression in infants carrying single common variant with combination of two common variants (*p* value = 0.42, one-way ANOVA analysis). Circles: outlier values/data; stars: extreme outlier values/data.

Based on risk analysis, the OR of all common variants identified (p.Gly71Arg, p.Pro229Gln, TA_5_, and TA_7_) were greater than one; however they were not significantly associated with the increased risk of NH in Deutromalay as the *p* value > 0.05 (Tables 1 and S6). Furthermore, STB levels of infants with TA_5_ and TA_7_ were not significantly different from infants carrying TA_6_ (major allele); this strengthens the fact that neither TA_5_ nor TA_7_ was associated with NH in this study. We further analyzed the differences of STB levels of infants carrying combination of TA repeats with common variants in the coding region (TA_6/7_+p.Pro229Gln, TA_6/6_+p.Gly71Arg, or TA_6/5_+p.Gly71Arg). However, the STB levels of infants carrying those combinations of common variants were not significantly higher than those with only TA repeats counterpart variants (Figure 4). Taken together, our results showed that different* UGT1A1 *genotypes did not significantly affect the severity of NH in the Indonesian population.

## 4. Discussion

In this study, we reported the first* UGT1A1* rare and common variants identified in infants with NH in Deutromalay population in Indonesia. Ten and four rare variants (MAF < 0.01) were identified in cases and controls, respectively. p.Gly71Arg and p.Pro299Gln variants, as in other Asian and Caucasian populations, are also common variants in Deutromalay population in Indonesia. Although the OR of p.Gly71Arg, p.Pro229Gln, and TA_7_ are above one, none of their *p* value is <0.05. These data show that none of those common variants is a risk allele for NH in Deutromalay population in Indonesia (Tables 1 and S6). Study by Yu et al. showed that TA_7_ and p.Gly71Arg are associated with the increased risk of NH in Asian population observed in meta-analysis, in which more than 6520 participants were included in the study [15]. Two hundred thirty-one participants (116 cases and 115 controls) included in this study most likely do not have enough power to detect those associations.

Of 10 rare variants identified in cases, 6 have been reported in the previous studies to be causally related to CN1 and CN2, while 4 of the identified remaining rare variants were novel (p.Ala61Thr, p.His300Arg, p.Lys407Asn, and p.Tyr514Asn) [10, 29]. Compound heterozygous p.Tyr486Asp with p.Gly71Arg and homozygous p.Gly71Arg have been identified in CN2 patients in different populations across Asia [29]. Instead with p.Gly71Arg, in this study, we identified a heterozygous compound of p.Tyr486Asp with deletion of TA sequence (TA_5_) in the promoter of one patient (ID79) (Table 2). It has been reported that the glucuronidation activity of the enzyme UGT1A1 containing p.Tyr486Asp reduces to only 10% of the wild type counterpart, while the TA_5_ variant is known to increase the* UGT1A1* expression [29]. If these two variants, which have contradictive effect on enzyme activities and gene expression, are located in different strand of DNA (*trans*-coinheritance) this might explain why this patient had a milder phenotype (STB level ~ 18.26 mg/dL) compared to that of CN1 patients (STB level ~ 30–50 mg/dL) which is more similar to that of CN2 patients (STB level ~6–20 mg/dL) [26, 29]. The same reason might be applied to other patients who have compounding heterozygous missense rare variant (p.His300Arg, p.Pro364Leu, p.His487Tyr, and p. Pro451Leu) with heterozygous deletion of TA sequence (TA_5_) variant in the promoter region (Table 2). Compared to the reported rare variants identified in CN1 and CN2 patients which are usually homozygous or compounding heterozygous variants in the coding region, there was a trend in this study that rare coding variants identified in cases were heterozygous and almost always present in combination with deletion of TA sequence (TA_5_) in the promoter (Table 2). One patient (ID 44) has a combination of three common variants (p.Gly71Arg, p.Pro229Gln, and TA_7_) and another patient (ID 178) has a combination of two heterozygous novel rare coding variants (p.Lys407Asn and p.Tyr514Asn) with the heterozygous common variant (p.Gly71Arg). Although the phenotypes of these two infants were not severe (STB levels were 19.3 and 20.7 mg/dL) as CN1, genetic counseling for their families might be required as the pattern of inheritance could be autosomal recessive (Crigler-Najjar syndrome).

Study by Kadakol et al., 2000, showed that the combination of TA_7_ variant in the promoter of* UGT1A1* in* trans-coinheritance* with structural (coding) variants could enhance the pathogenicity of the coding variant, as the TA_7_ variant decreases the expression of wild type UGT1A1 protein [30]. Study by Maruo et al., 2016 showed that different combination of rare and common variants resulted in different phenotype, starting from mild to severe, of NH and hyperbilirubinemia in adults [26]. Those studies showed that different* UGT1A1 *genotypes (combination of common and rare variants) affect the severity of the disease. In this study, we could not group the cases based on combination of rare and common variants, as most of the rare variants present only in one patient. If we group the genotype of patients based on combination of common variants, we observed that the STB level of infants with only TA repeat variant and those who have combination of TA repeat and coding common variant (such as p.Gly71Arg or p.Prof229Gln) did not significantly differ (Figure 3). In conclusion, different combination of* UGT1A1 *common variants (genotypes) observed in this study did not significantly affect the severity of NH phenotype in Indonesian population. In terms of genotype-phenotype correlation, we observed that infants with rare variants located in exon 5 (p.Pro451Leu, p.Tyr486Asp, p.His487Tyr, and p.Tyr514Asn) have STB level higher than 18 mg/dL (Table 2). Those variants are located close to the transmembrane domain (Figure 1); this might affect protein internalization into the membrane of endoplasmic reticulum (ER) and nuclear envelope in the hepatocyte cell and hence disturb its function. It is interesting to mention that in this population heterozygous p.Pro229Gln variant presents always in combination with TA_7_ allele. Similar result had been reported in CN2 and Gilbert syndrome patients in Japanese population and in neonatal hyperbilirubinemia in Chinese population [25, 26]. This might indicate that these two variants are in the linkage disequilibrium in Asian population.

Common and rare variants were identified in controls as well; although not significant (*p* = 0.07) more infants in cases (5.17%) are carrying rare variants compared to those in controls (1.7%) (Tables 1 and 2). One of those rare variants identified in controls was a variant caused a premature stop codon (p.Tyr79X). This variant supposedly leads to a truncated protein and hence most likely is pathogenic. However surprisingly this infant did not experience severe hyperbilirubinemia (STB level is 6.99 mg/dL). The possible explanation for this is that either the wild type allele alone could cover the overall UGT1A1 function or this rare variant, for unknown reason, did not lead to premature stop-gain function. Interestingly, there was a stop-gain variant identified in CN1 patient proven to induce skipping of exon-containing variant rather than cause premature stop codon in hepatic mRNA [31]. This rare variant produced an in-frame shorter protein which is still functioning. This could be a possible explanation why sometimes the predicting pathogenic stop-gain variants do not lead to severe phenotype. Whether or not the same mechanism applies on the p.Tyr79X variant still needs further studies.

A remark that needs to be treated with caution is that one needs to consider many aspects, including the nature of the disease itself, to conclude whether or not rare variant identified is pathogenic. Neonatal hyperbilirubinemia (NH), for example, is considered as a polygenic and multifactorial complex in nature. Many variants in many different genes might contribute in concert to induce NH. In this study, four subjects in the controls group from whom* UGT1A1* rare variant was identified had STB level > 6 mg/dL and even two of them had STB level > 12 mg/dL. This shows that although the phenotype is not as severe as those in cases groups, still those rare variants might contribute to the high STB level in those subjects. Adding specific inducer (e.g., breast feeding) might lead them to have NH phenotype.

Haplotype analysis showed that in this study a single haplotype containing major alleles of rs8830, rs10929303, and rs1042640 (CCC) located in 3′ UTRS was significantly associated with neonatal hyperbilirubinemia (*p* = 0.0025) (Table 4). Those SNPs located in 3′ UTRs are usually involved in the mRNA stability. The major alleles of those SNP most likely stabilize the* UGT1A1* mRNA; hence one could assume that this haplotype might modify the risk of getting NH if it is in* cis*-position with pathogenic* UGT1A1* coding variants. However, study by Court et al., 2013, showed that the minor alleles of those SNPs did not affect the mRNA stability but created exon splice enhancer which favored certain splicing site on exon 5 (exon 5a) [32]. Whether or not this alternative splicing could reduce the risk of developing hyperbilirubinemia in infants needs further study.

## 5. Conclusion

Our study provides information that* UGT1A1* rare variants are identified more frequently in cases (5.17%) than in controls (1.70%) in Indonesian population. Combination of common variants located in the promoter (TA6, TA7, and T5) within the coding region of* UGT1A1* (p.Gly71Arg or p.Pro229Gln) did not significantly affect the STB levels and hence did not contribute to the severity of NH phenotype in Indonesian population.

## Figures and Tables

**Table 1:** UGT1A1 (NM_000463) variants identified in cases and controls.

Number	Nucleotide change	Amino acid change	SNP 142	Type of variant	MAF dbSNP138	Cases (individual/allele/MAF)	Controls (individual/allele/MAF)	OR	95% CI	*p* value
(1)	c.141C>T	p.Ile47=	rs34526305	Silence	0.0010	1/1/0.0043	-	-	-	-
(2)	c.211G>A	p.Gly71Arg	rs4148323	Missense	0.0343	16/16/0.068	11/11/0.047	1.47	0.67–3.25	0.33
(3)	c.686C>A	p.Pro229Gln^#^	rs35350960	Missense	0.0028	11/11/0.047	6/7/0.030	1.59	0.60–4.16	0.35
(4)	c.1091C>T	p.Pro364Leu	rs34946978	Missense	0.0022	2/2/0.0086	-	-	-	-
(5)	c.1456T>G	p.Tyr486Asp	rs34993780	Missense	0.0008	1/1/0.0043	-	-	-	-
(6)	c.1459C>T	p.His487Tyr	rs371183955	Missense	0.0004	2/2/0.0086	1/1/0.0043	-	-	-
(7)	c.1352C>T	p.Pro451Leu	rs114982090	Missense	0.0014	1/1/0.0043	-	-	-	-
(8)	c.237C>G	p.Arg336Trp	rs139607673	Missense	NA	1/1/0.0043	-	-	-	-
(9)	c.899A>G	p.His300Arg^∗^	NA	Missense	NA	1/1/0.0043	-	-	-	-
(10)	c.181G>A	p.Ala61Thr^∗^	NA	Missense	NA	1/1/0.0043	-	-	-	-
(11)	c.1221G>T	p.Lys407Asn^∗^	NA	Missense	NA	1/1/0.0043	-	-	-	-
(12)	c.1540T>A	p.Tyr514Asn^∗^	NA	Missense	NA	1/1/0.0043	-	-	-	-
(13)	c.237C>G	p.Tyr79X^∗^	NA	Stop-gain	NA	-	1/1/0.0043	-	-	-
(14)	c.1037C>T	p.Ala346Val^∗^	NA	Missense	NA	-	1/1/0.0043	-	-	-
(15)	c.1234A>T	p.Thr412Ser^∗^	NA	Missense	NA	-	1/1/0.0043	-	-	-

*Total Individual *						*39*	*21*			

^#^Considered as common variant in Deutromalay population. ^*∗*^Novel variant. NA: not available.

**Table 2:** Common and rare variants identified in cases and controls and in silico analysis.

Number	ID	Rare variants	Exon	Genotype (Ref/Alt)	TA repeat (*n*/*n*)	Common variants	Genotype (Ref/Alt)	STB (mg/dL)	In silico analysis of mutations		
									PolyPhen-2	SIFT	Mutation Taster
*Cases*											
							
(1)	37	p.Ala61Thr^*∗*^	1	G/A	6/5	-	-	23.27	B	T	P
(2)	28	p.His300Arg^*∗*^	2	A/G	6/5	-	-	14.81	PD	D	DC
(3)	44	-	-	-	6/7	p.Gly71Arg	G/A	20.74	-	-	-
						p.Pro229Gln	C/A				
(4)	72	p.Ile47=	1	C/T	6/5	-	-	18.12	-	D	-
(5)	76	p.Arg336Trp	3	C/T	6/7	p.Gly71Arg	G/A	15.10	PD	D	DC
(6)	77	p.Pro364Leu	4	C/T	6/5	-	-	26.98	PD	D	DC
(7)	43							17.44			
(8)	11	p.His487Tyr	5	C/T	6/5	-	-	20.59	PD	D	P
(9)	86							19.55			
(10)	33	p.Pro451Leu	5	C/T	6/5	-	-	25.77	PD	D	DC
(11)	79	p.Tyr486Asp	5	T/G	6/5	-	-	18.26	PD	D	
(12)	178	p.Lys407Asn^*∗*^	4	G/T	6/6	p.Gly71Arg	G/A	19.38	PS	D	P
(13)		p.Tyr514Asn^*∗*^	5	T/A					PS	D	P
*Controls*											
								
(1)	200	p.Tyr79X^*∗*^	1	C/G	6/7	-	-	6.99	-	D	-
(2)	101	p.Ala346Val^*∗*^	4	C/T	6/6	-	-	12.81	PD	D	P
(3)	263	p.Thr412Ser^*∗*^	4	A/T	6/6	-	-	13.75	B	T	P
(4)	232	p.His487Tyr	5	C/T	6/7	-	-	6.8	PD	D	P

Ref: reference allele; Alt: alternative allele. ^**∗**^Novel variant. B: benign, PD: probably damaging, PS: possibly damaging, T: tolerated, D: damaging, P: polymorphism, DC: disease causing, and STB: serum total bilirubin.

**Table 3:** Association analysis of identified common variants.

Number	SNP	Coordinate Hg19	Ref	Alt	F_A	F_U	CHISQ	*p* value	OR	CI (low-up)	*q*-value
(1)	rs10929303	234681416	C	T	0.078	0.158	7.0800	**0.008**	0.4521	0.1946–1.0480	**0.062**
(2)	rs8330	234681645	C	G	0.078	0.141	4.6710	0.030	0.5172	0.2431–1.3864	0.112
(3)	rs1042640	234681544	C	G	0.078	0.136	4.1270	0.042	0.5360	0.2337–1.3176	0.112
(4)	rs2302538	234676413	T	C	0.091	0.051	2.8120	0.093	1.8590	0.6560–5.3130	0.187
(5)	rs4148323	234669144	G	A	0.078	0.051	1.3960	0.237	1.5710	0.5402–4.6176	0.379

Ref: reference, Alt: alternative, F_A: frequency Alt in cases, and F_U: frequency Alt in controls.

**Table 4:** Haplotypes analysis (rs8330 (C>G), rs10929303 (C>T), and rs1042640 (C>G)).

Haplotype	Frequency		Chi square	*p* value
	Cases	Controls		
CCC	0.922	0.829	9.111	**0.0025**
GTG	0.078	0.128	3.117	0.0775
CTC	0.000	0.030	6.971	**0.0083**

## Supplementary Materials

Supplementary Materials Table S1: list of primers used for amplified exons region of UGT1A1. Table S2: characteristic data of neonates in cases and controls group. Table S3: characteristics of the mothers. Table S4: results of Complete Blood Count (CBC) test of neonates. Table S5: summary of statistics of deep-targeted sequencing results. Table S6: risk analysis of TA repeats in the promoter of UGT1A1.
