# Supplementary material for: UGT1A1 Genetic Variations and a Haplotype Associated with Neonatal Hyperbilirubinemia in Indonesian Population
Source: Biomed Res Int. 2018 Jan 23;2018:9425843. doi: 10.1155/2018/9425843 (PMC5828093; doi:10.1155/2018/9425843)
Supplement: Supplementary Materials — Table S1: list of primers used for amplified exons region of UGT1A1. Table S2: characteristic data of neonates in cases and controls group. Table S3: characteristics of the mothers. Table S4: results of Complete Blood Count (CBC) test of neonates. Table S5: summary of statistics of deep-targeted sequencing results. Table S6: risk analysis of TA repeats in the promoter of UGT1A1. [file 9425843.f1.pdf]

SUPPLEMENTARIES

Table S1 – Primers for Exons of *UGT1A1* (NM_000463)

Exon	Variant	Primer	Sequences 5’ → 3’	Product Size (bp)
1	p.I47=	UGT1A1_1_F	TCTCTGAAAGTGAACTCCCTGC	700
		UGT1A1_1_R	CTCAAATTCCAGGCTGCATGG	
3	p.Arg336Trp<br>p.Arg346Val	UGT1A1_3_F	GCATCCACTTGTTTCATTAAGC	415
		UGT1A1_3_R	ACCTATACATCCAATCCGCCC	
5	p.His487Tyr<br>p.Tyr486Asp	UGT1A1_5_F	TCTGGTAGTCTTCTTAAGCAGCC	446
		UGT1A1_5_R	AGTTTGGAAATGACTAGGGAATGG	

Table S2: Characteristic of Neonates in Cases and Controls Groups

Characteristic	Cases (N=116)	%	Controls (N=115)	%	p-value*
**Sex**					**0,950**
Male	61	52.6	60	52,2	
Female	55	47.4	55	47,8	
**Birth Weight (gram)**					**0,772**
Average (SD)	3125 (345,6)		3138 (371,2)		
Range	2.500−4.300		2.500−4.250		
**Feeding**					**0,483**
Breast milk	94	81,0	98	85,2	
Formula	-	-	-	-	
Mix of both	22	19,0	17	14,3	
**Sibling with Jaundice**					**0,319**
Yes	26	22,4	19	16,4	
No	90	77,6	96	80,6	
**ABO Blood Group**	18	15,5	9	7,75	**0,065**
**Coombs Test Results**					
Negative	116	100	115	100	
Positive	-		-0		

*) Chi–square, except Birth weight

Table S3 - Characteristic of the Mothers

Characteristic	Cases (n=116)	%	Controls (n=115)	%
**Age (Years)**				
Average (SD)	30 (6,1)	-	30,9 (5,8)	-
Median	30	-	31	-
Range	18–43	-	17–47	-
**Parity**				
1	54	46.6	41	35.7
2–3	50	43.1	61	53.0
≥ 4	12	10.3	13	11.3
Median	2	1.7	2	1.7
Range	1–7	-	1–7	-
**Delivery method**				
Cesarean delivery	57	49.1	87	75.7
Normal	53	45.7	28	24.3
Vacuum	3	2.6	0	0
Forceps	3	2.6	0	0
**Consanguinity**				
Yes	0	0	0	0
No	116	100	115	100

Table S4 –Results of Complete Blood Count (CBC) Test

	Cases (n=116)	Controls (n=115)
**Hemoglobin (g/dL)**		
Average (SD)	16.52 (2.5)	15.91 (2.0)
Range	9.5–22.90	11.6–21.6
**Hematocrit (%)**		
Average (SD)	46.04 (7.72)	44.68 (5.67)
Median	46.65	43.65
Range	27.0–63.10	31.7–59.6
**Leukocyte (µL)**		
Average (SD)	12.83 (3.60)	15.52 (5.20)
Median	12.47	15.22
Range	2.9–24.8	3.7–32.3
**Reticulocyte**		
Average (SD)	3.55 (2.86)	4.40 (2.44)
Median	2.94	4.65
Range	0.45–14.8	0.52–10.73
**Erythrocyte**		
Average (SD)	4.87 (1.12)	4.62 (0.59)
Median	4.79	4.56
Range	2.69–13.40	3.49–6.35

Table S5 - Statistic of Deep-Targeted Sequencing Results

	**Cases N (%)**	**Controls N (%)**
Coverage (Average)		
Variants Identified	521	498
Variants with coverage ≥ 10	481	448
Deletion	37(8)	26(6)
Insertion	21(4)	21(5)
SNP	423(88)	401(89)
* UTRs	344	349
* Intronic	38	31
* Exonic	40	21
* Downstream of the gene	1	0

SNP : Single Nucleotide Polymorphism, UTRs: Untranslated Regions

Table S6 – Risk Analysis of TA repeats in the promoter of *UGT1A1*

**Allele**	**Cases (n=232)**		**Controls (n=230)**		**OR**	**95%CIs**	**p-value***
	**n**	**%**	**n**	**%**			
TA_6	149	64.2	157	68.3			
TA_5	58	25.0	51	22.2	1.19	0.77-1.86	0.41
TA_7	25	10.8	22	9.5	1.20	0.65-2.22	0.57

*Chi−Square
